# Molecular identification of *Borrelia* and SFG *Rickettsia* spp. in hard ticks parasitizing domestic and wild animals in southeastern Spain

**DOI:** 10.1007/s11259-023-10292-x

**Published:** 2024-01-17

**Authors:** N. Ortega, I. Arcenillas-Hernández, MI Villa, MD González, MR Caro

**Affiliations:** 1 Dpto. de Sanidad Animal, Facultad de Veterinaria, Campus de Excelencia Internacional, “Campus Mare Nostrum” Universidad de Murcia, Espinardo, Murcia, 30100 Spain; 2 Dirección General de Salud Pública y Adicciones. Servicio de Seguridad Alimentaria y Zoonosis, Consejería de Salud de la Comunidad Autónoma de Murcia, Murcia, Spain

**Keywords:** Mediterranean basin, Emerging Disease, One health, Ixodidae

## Abstract

Lyme disease and the spotted fever group rickettsiosis, involve bacteria belonging to the genus *Borrelia* and *Rickettsia*, respectively. These infections are the most important tick-borne zoonotic diseases involving ticks as vectors. Descriptive and epidemiological studies are essential to determine the animal hosts involved in the maintenance of these diseases. In the present study, 94 tick pool samples from 15 different host species located in the Region of Murcia (southeastern, Spain) were analysed. Ticks were morphologically identified as: *Dermacentor marginatus, Hyalomma lusitanicum, Ixodes Ricinus*, and *Rhipicephalus sanguineus*. Our results showed that 5.3% of the tick pool samples carried *Borrelia* spp. DNA, and 20.2% carried SFG *Rickettsia* DNA. In every hard tick pool Spot Fever Group (SFG) *Rickettsia* spp. DNA were detected, except for *H. lusitanicum.* Likewise, *D. marginatum* was the only species in which *Borrelia* spp. DNA was not detected. Barbary sheep and wild boar were the host species in which tick pools showed DNA presence of both pathogens. This study increases the knowledge about the presence of *Borrelia* spp. DNA and SFG *Rickettsia* spp. DNA in different hard tick species from this geographical area.

**Supplementary Information:**

The online version contains supplementary material available at 10.1007/s11259-023-10292-x.

## Introduction

Emerging tick-borne zoonotic diseases are a major global public health risk that has been increasing in recent decades (Parola et al. 2013). Ticks can act as vectors of dangerous bacterial, parasitic, and viral zoonotic pathogens, so epidemiologic studies in them are essential to identify zoonotic agents and their host animals in different geographical areas.

Lyme disease (LD) is one of the most prevalent zoonotic tick-borne infectious diseases caused by species or members of the *Borrelia burgdorferi* sensu lato (Bbsl) (Wolcott et al. 2021). The main vector is *Ixodes ricinus*, widely distributed throughout the Iberian Peninsula, with a tendency to inhabit deciduous forests with shrubs. For an effective host-to-host transmission, a tick must acquire the bacteria from an infected animal, which allows it to survive and replicate while moulting, as well as be transmitted to another animal host during the next blood meal. The diversity of the *Borrelia* complex, which can cause human infection, is greater associated with *B. burgdorferi* sensu stricto (Bbss) species: *Borrelia afzelii, Borrelia valaisiana* and *Borrelia garinii*, being *B. afzelii* and *B. garinii* the most associated with human disease. This disease has an estimated global potential incidence of 12.3 million/year and a prevalence of 62.1 million cases, including a high percentage of undiagnosed and misdiagnosed cases due to failures and difficulties associated with diagnostic methods (Cook and Puri 2020).

Ticks are also considered the main vectors of SFG *Rickettsia* spp., being the main source of natural infection in tick-borne rickettsiosis (TBR) in Europe (Parola et al. 2013). In the Iberian Peninsula zoonotic species as *Rickettsia conorii*, *Rickettsia monacensis, Rickettsia massiliae, Rickettsia aeschlimannii, Rickettsia raoultii* or *Rickettsia slovaca* have been reported (Castillo-Contreras et al. 2021; Remesar et al. 2022). Animal host species can play an important role as carriers of these microorganisms in the environment (Parola et al. 2013), due to the increase of contact between domestic and wild species, and the human-wildlife interaction occurring in anthropized areas.

The aims of this study were to determine the DNA presence of Bbsl and SFG *Rickettsia* spp. in different hard tick species collected from domestic and wild animals in southeastern Spain.

## Materials and methods

### Study area, animals, and tick samples

A total of 463 ticks were collected from the Region of Murcia (SE, Spain) a semi-arid geographical area. Ninety-four tick pool samples (with 1–10 individuals/pool) were collected from 76 individuals of 15 different animal species (Table 1) during the spring (March-April) and autumn (September-October) seasons of 2022. Tick pools were made according to their species, without differentiating males (n = 195) from females (n = 268) or any life stage (340 were adults and 123 were nymphs). Ticks were properly removed with tweezers, and specimens were classified using a taxonomic key (Estrada-Peña et al. 2017). Finally, ticks were preserved in absolute ethanol until processing. Only ticks identified morphologically as genus *Ixodes* were additionally subjected to molecular identification by DNA Barcode, using for insect cell lines, the primers LepFl and LepRl (Cooper et al. 2007) these analyses were performed by the Molecular Biology Service (ACTI) of the University of Murcia (Spain).

### Molecular detection of Bbsl complex and SFG rickettsia spp

For DNA extraction ticks were pooled according to species and individual host and they were left overnight at room temperature to allow complete evaporation of the ethanol. Subsequently, Extracted Genomic DNA Tissue kit (BLIRT S.A. Germany) was used, following the manufacturer’s instructions. The extracted DNA was stored at -20ºC until their use.

Bbsl 23 S rRNA real-time qPCR was performed with Fast Gene Probe 2xqPCR Universal Mix (Nippon Genetics Europe GmbH) using the primers Bb23Sf and Bb23Sr and Bb23Sp-FAM probe (Courtney et al. 2004) to generate 75 bp fragment, PCR reactions were performed in duplicate and specific DNA from *B. burgdorferi* B31 strains and pure water were used as positive and negative controls, respectively.

Two different PCRs were used for genotypic identification of Rickettsiae, the first one was specific for the *gltA* gene and allows amplification of *Rickettsia* spp. 380–397 bp fragment, using the primers RpC5.877p and RpC5.1258n (Regnery et al. 1991), and Zestaw NXT Taq kit hot start PCR (EURx. Poland). Samples that tested positive for the *gltA* gene were used for a second PCR, specific for gene encoding the protein rOmpA that allows amplification of the SFG *Rickettsia* 629–632 bp fragment using primers Rr190.70p and Rr190.701n (Roux et al. 1996), and Zestaw NXT Taq kit hot start PCR (EURx. Poland). For both PCR, specific DNA from SFG *Rickettsia* spp. and pure water were used as positive and negative controls, respectively. PCR products are analysed by gel electrophoresis on a 1.5% agarose gel stained with Midori Green (Nippon Genetics).

For the nucleotide sequencing study 19 PCR-amplified Rickettsiae gene protein rOmpA sequences and 5 PCR-amplified *B. burgdorferi* 23 S rRNA sequences were selected and used. Amplicons of the expected size were sequenced on an Applied Biosystems 3500 Genetic Analyzer following the manufacturer’s protocol. The sequences obtained were aligned with MEGA11: Molecular Evolutionary Genetics Analysis version 11 (Tamura et al. 2021) and compared with those sequences of other *Rickettsia* and *Borrelia* species downloaded from the GenBank database to identify known sequences with a high degree of similarity.

### Statistical analysis

R software 3.3.0 was used to statistical analysis, where Fisher’s test was used to evaluate statistical differences between tick pools species including in our study, considering as a significant difference the threshold *p* < 0.05.

## Results

**Fig. 1:**
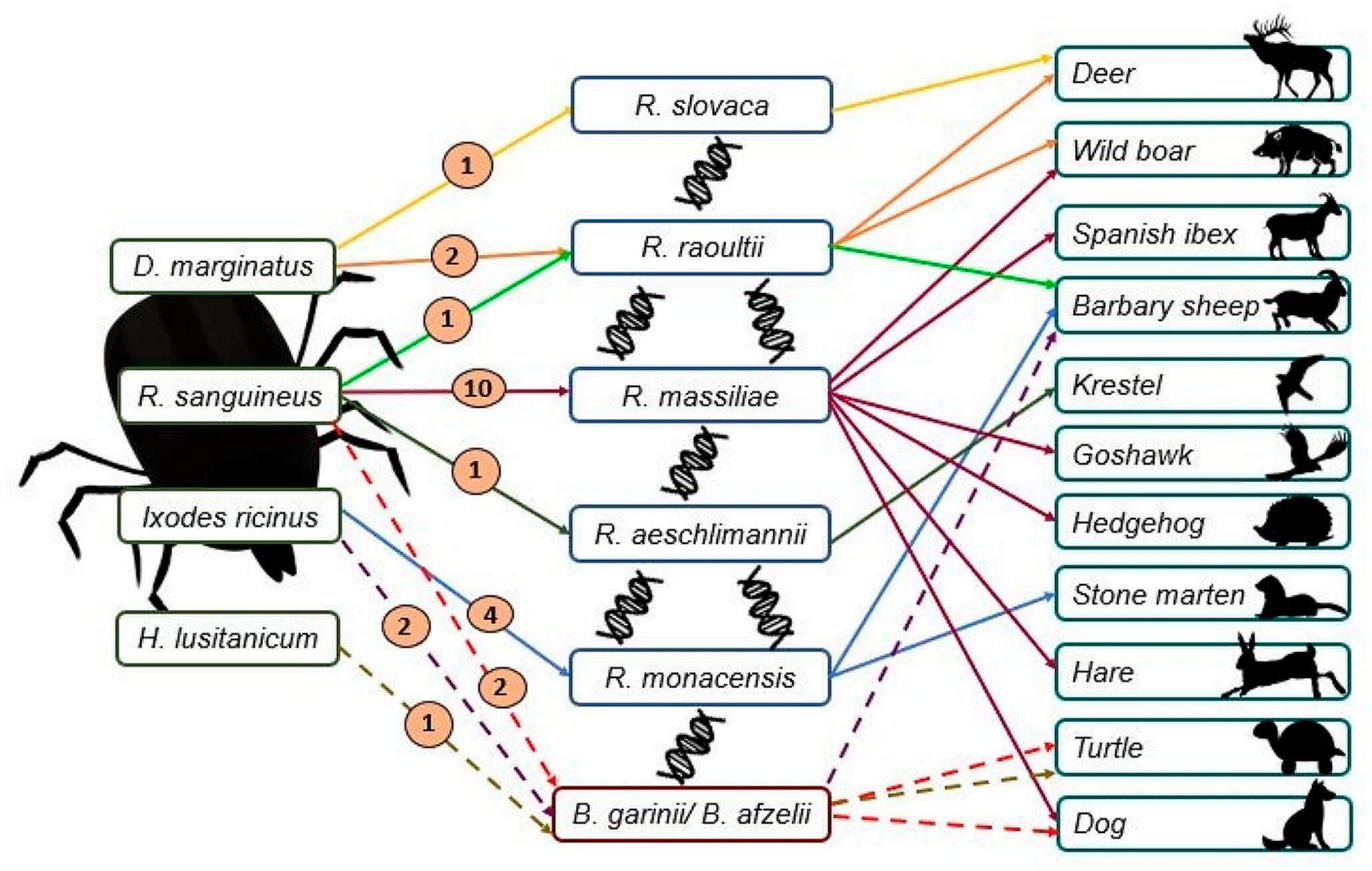
SFG *Rickettsia* and *Borrelia* species identified in different pools of tick species and the host from which they were extracted. Number of pools used for each tick species is included

Four different tick species: *Dermacentor marginatus, Hyalomma lusitanicum*, *Ixodes ricinus* and *Rhipicephalus sanguineus*, were identified. *Borrelia* spp. DNA were detected from 5.3% of tick pools (5/94; 95%CI 0.7–9.8): 2/5 from *I. ricinus*, 2/5 from *R. sanguineus*, and 1/5 from *H. lusitanicum* collected from 2 barbary sheep, one Mediterranean tortoise, and one dog, respectively (Table 1; Figure S1). Sequencing analysis of the 75 bp fragment identified: *B. garinii* (CP024316) and *B. afzelii* (CP075440), reaching 100% homology in all cases (Fig. 1; Table S1). *Rickettsia* spp. DNA were detected from 48.9% of tick pools (46/94; 95%CI 38.8–59.0), and specifically 41.3% of them (19/46; 95%CI 27.1–55.5) corresponding to DNA of SFG *Rickettsia* species; being present in tick pools of the majority of animal species sampled, except for Mediterranean tortoise, sheep, red fox, and eagle (Table 1) and mostly associated with *R. sanguineus* pools (Table 2). Sequence analysis identify five different SFG *Rickettsia* species: *R. massiliae* (KR401146, DQ212707 and KU498298), *R. raoultii* (KX506737, MT321626 and MH932055), *R. slovaca* (MF379311), *R. monacensis* (MK922659 and MN853333), and *R. aeschlimanni* (MF379306) (Fig. 1; Table S1). The homology between sequences ranged from 99.6 to 100%.

The concurrent DNA presence of *Borrelia* spp. and SFG *Rickettsia* spp. were detected in tick pools from 3.2% animals (3/94; 95%CI -0.4-6.7): two pools of *I. ricinus* collected from two barbary sheep, respectively, and one pool of *R. sanguineus* from a dog.

**Table 1:** Number of host animal species (N), number of total tick pools (with 1–10 ticks/pool), tick species and DNA detection (with number of positive pools (n))

Animal host species (N)	Total tick pools	Tick species^(1)^	DNA detection^(2)^ (n)
Golden eagle (*Aquila chrysaetos*) (1)	1	Hl	R (1)
Goshawk (*Accipiter gentilis*) (1)	1	Rs	R (1); SFG (1)
Barbary sheep (*Ammotragus lervia*) (19)	1	Hl	-
	16	Rs	R (7); SFG (2)
	10	Ir	R (3); SFG (3); B (2)
Spanish ibex (*Capra pyrenaica*) (7)	7	Rs	R (5); SFG (1)
Common kestrel (*Falco tinnunculus*) (1)	1	Rs	R (1); SFG (1)
Deer (*Cervus elaphus*) (3)	2	Dm	R (2); SFG (2)
	2	Rs	R (1)
European hedgehog (*Erinaceus europaeus*) (3)	2	Hl	R (2)
	3	Rs	R (3); SFG (3)
Rabbit (*Oryctolagus cuniculus*) (1)	1	Rs	-
Stone marten (*Martes foina*) (1)	1	Ir	R (1); SFG (1)
Mediterranean tortoise (*Testudo hermanni*) (1)	1	Hl	R (1); B (1)
	1	Rs	B (1)
Wild boar (*Sus scrofa*) (18)	18	Hl	R (4)
	3	Dm	R (2); SFG (1)
	2	Rs	R (1); SFG (1)
Hare (*Lepus europaeus*) (1)	1	Rs	R (1); SFG (1)
Sheep (*Ovis aries*) (1)	1	Hl	R (1)
Red fox (*Vulpes vulpes*) (4)	2	Rs	-
	2	Ir	R (1)
Dog (*Canis lupus familiaris*) (14)	1	Hl	-
	14	Rs	R (7); SFG (2); B (1)
Total (76)	94		

^(1)^ Rs: *Rhipicephalus sanguineus*; Hl: *Hyalomma lusitanicum*; Dm: *Dermacentor marginatus*; Ir: *Ixodes ricinus*. ^(2)^B: *Borrelia* spp, R: *Ricketts*i.a. spp., SFG: SFG *Rickettsia* spp)

**Table 2:** Percentage of *Rickettsia* spp., SFG *Rickettsia* spp. and *Borrelia* spp. tick pools with DNA presence based on the different tick species identified

		Percentage of tick pools (%)		
Tick species	Total tick pools	*Rickettsia* spp.	SFG *Rickettsia*	*Borrelia* spp.
*R. sanguineus*	51	52.9% (28/51)	23.5% (12/51)	3.9% (2/51)
*H. lusitanicum*	25	36% (9/25)	0	4% (1/25)
*D. marginatus*	5	80% (4/5)	60% (3/5)^(*)^	0
*I. ricinus*	13	38.4% (5/13)	30.7% (4/13)	15.4% (2/13)
Total	94	48.9% (46/94)	20.2% (19/94)	5.3% (5/94)

^(*)^ Statistically significant differences (*p* < 0.05)

## Discussion

The findings described in this study suggest the presence of *Borrelia* an SFG *Rickettsia* in natural and urban environments in southeastern Spain. Although the detection of a microorganism in a tick collected on an animal do not demonstrate the vector capacity of this tick species, some of the results found in this study have been quite novel, either due to the species of tick where the DNA is detected or because the host species in which these ticks were found.

All tick species detected in this study had been previously identified in the Iberian Peninsula (Estrada-Peña et al. 2017; Remesar et al. 2022). *Rhipicephalus sanguineus* and *H. lusitanicum* are generally present in semi-arid environments and are the most abundant tick species in this study (Estrada-Peña et al. 2017). Indeed, *H. lusitanicum*, has suffered an increase in its population along northeastern Spain (Castillo-Contreras et al. 2021). This species could also be spreading throughout southeastern Spain, since in this study *H. lusitanicum* was the second most abundant tick species.

Interestingly, our results also showed the presence of *I*. *ricinus* and *D. marginatus*, ticks frequently associated with more humid environments (Estrada-Peña et al. 2017). Although *Ixodes* spp. preferably lives in moist environments with cover vegetation that helps to maintain humidity during warmer periods, their presence in our studied territory may be associated to the scrub or wooded areas available that could create an optimal microclimatic condition that favour the occurrence of this tick (Kahl and Gray 2023).

In the life cycle of *Borrelia*, larvae and nymphs feed on small mammals, but large mammals and reptiles are used as primary hosts for mating adults to maintain tick population (Gern et al. 1998). This is consistent with our results, as tortoise, dog and barbary sheep were the host species in which positive *Borrelia* spp. DNA tick pools were detected. Results from barbary sheep were the most expected, as this ungulate species tends to inhabit areas with denser vegetation cover and, therefore, are more likely to encounter *Ixodes spp.* ticks. Our sequencing data, performed on a fragment of only 75 bp, showed that *Borrelia* species detected in this study could be either *B. garinii* or *B. afzelii*, it was not possible to discriminate between the two species based on the small size of the amplified fragment. However, both species are related to LD in the European countries (Mendoza-Roldan et al. 2019). In the Mediterranean basin, the most notable prevalences have been associated with *B. lusitaneae* where certain lizard species haven been identified as the main reservoir host of this species (Dsouli et al. 2006; Norte et al. 2015). Although our study did not detect this species or include samples from lizards, *B. garinii* or *B. afzelii* have been detected in another reptile, the Mediterranean tortoise, so further research would be interesting to evaluate the presence of Bbsl in different reptile species in this region. Our finding could explain the increasing LD cases reported in the Region of Murcia, where LD incidence by *Borrelia* spp. has been triplicated in the last 15 years in this region, being one of the Spanish regions where a greater increase has been observed since 2007 (238%) (Amores et al. 2022).

Moreover, SFG *Rickettsia* DNA was detected in 30.7% of *I*. *ricinus* tick pools of this study. A similar rate was reported in a study conducted in northern Spain (40%; 10/25) (Remesar et al. 2023). Nevertheless, our percentage was higher compared with other European studies carried out, for example, in Italy (14%; 30/215) (Cafiso et al. 2021) or France (1.4%; 10/696) (Akl et al. 2019). The differences found between these studies could be explained by the bias caused by the low number of ticks analysed. *Rickettsia monacensis* was the only SFG *Rickettsia* species identified from *I*. *ricinus* tick pools, and this same association has also been recently reported in a French study (Akl et al. 2019). Therefore, *I. ricinus* presence should be considered by the health authorities since it may represent an epidemiological risk.

Despite the low number of ticks pools in our study, *D. marginatus* was the tick species in which SFG *Rickettsia* DNA was mostly detected and the only tick species with a statistically significant result. A similar percentage was reported in Barcelona (NE, Spain) by Castillo-Contreras et al. (2021), while other studies in natural and urban environments in Italy obtained a significantly lower frequency (Sgroi et al. 2020). In accordance with other studies conducted in Europe (Pereira et al. 2018; Sgroi et al. 2020), D. *marginatus* ticks from host species such as deer and wild boar, showed in this study DNA of two different *Rickettsia* species: *R. slovaca* and *R. raoultii*.

The most frequent *Rickettsia* species detected in this study was *R. massiliae*, which was only detected in *R. sanguineus*. This was also reported in Italian ticks collected from wild carnivores (Chisu et al. 2017). *Rickettsia raoultii* and *R. aeschlimannii*, were also sequenced from *R. sanguineus* ticks in our study, and they have been previously reported from southern Europe areas, including the Iberian Peninsula (Chisu et al. 2017; Pereira et al. 2018; Castillo-Contreras et al. 2021). *Hyalomma* spp. ticks have an important contribution to the maintenance of *R. aeschlimannii* in natural environments. Nevertheless, in our case, no DNA SFG *Rickettsia* species were detected in this tick species. In this sense, other studies conducted in Spain showed similar results suggesting that *H. lusitanicum* might not act as an optimal vector of *Rickettsia* species (Pereira et al. 2018; Castillo-Contreras et al. 2021).

Our results suggest that wild boar may be contributing to the dispersal and/or maintenance of *D. marginatus* and *H. lusitanicum* ticks in urban and peri-urban environments, increasing the risk of zoonotic diseases transmission (Sgroi et al. 2020; Castillo-Contreras et al. 2021).

The approach of wildlife to peripheral urban areas caused by habitat fragmentation has establish an interaction between wildlife, domestic animals, and humans (Cafiso et al. 2021) and could increase the risk of disease transmission in the wild-domestic-human interface. Hence, combined analysis considering environmental and epidemiological factors are essential to develop effective control strategies for pathogens such as SFG *Rickettsia* species or *Borrelia* spp.

## Conclusions

DNA of both pathogens, SFG *Rickettsia* spp. and *Borrelia* spp. have been detected in *I*. *ricinu*s and *R. sanguineu*s ticks so they could be acting as carriers of both pathogens in the southeastern of Spain. In terms of host species, Bbsl was detected in *H. lusitanicum* and *R. sanguineus*, both ticks from a Mediterranean tortoise. This is the first time that *Borrelia* has been detected in this reptile in the studied area. In addition, this study provides information about the presence of *I. ricinus* that are not usually present in semi-arid environments. In this sense, further studies would be necessary for a better understanding of emerging tick-borne diseases in Spain, as well as the tick species circulating in this territory.

### Electronic supplementary material

Below is the link to the electronic supplementary material.

Supplementary Material 1

Supplementary Material 2

## Data Availability

The datasets generated during and/or analysed during the current study are available from the corresponding author on reasonable request.
