# Supplementary figures and images for: Molecular identification of Borrelia and SFG Rickettsia spp. in hard ticks parasitizing domestic and wild animals in southeastern Spain
Source: Vet Res Commun. 2024 Jan 17;48(3):1785–90. doi: 10.1007/s11259-023-10292-x (PMC11147859; doi:10.1007/s11259-023-10292-x)

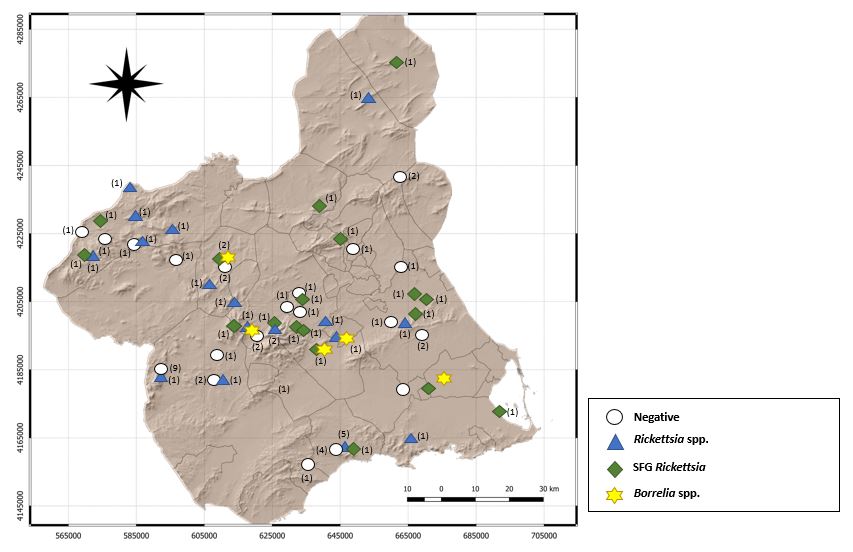

Supplement: Supplementary file 1 — Supplementary Material 1 [file 11259_2023_10292_MOESM1_ESM.jpg]
