# Supplementary material for: Molecular identification of Borrelia and SFG Rickettsia spp. in hard ticks parasitizing domestic and wild animals in southeastern Spain
Source: Vet Res Commun. 2024 Jan 17;48(3):1785–90. doi: 10.1007/s11259-023-10292-x (PMC11147859; doi:10.1007/s11259-023-10292-x)
Supplement: Supplementary file 2 — Supplementary Material 2 [file 11259_2023_10292_MOESM2_ESM.docx]

Table S1: Supplementary material with sequencing analysis results

| **Host species** | **Tick species** | **SFG *Rickettsia* species** | **Homology (%)** |
| --- | --- | --- | --- |
| Deer | *D. marginatus* | *R. slovaca* | 100% |
| Wild boar |  | *R. raoultii* | 100% |
| Deer |  | *R. raoultii* | 100% |
| Stone marten | *I. ricinus* | *R. monacensis* | 100% |
| Barbary sheep |  | *R. monacensis* | 100% |
| Barbary sheep |  | *R. monacensis* | 99.8% |
| Barbary sheep |  | *R. monacensis* | 100% |
| Hedgehog | *R. sanguineus* | *R. massiliae* | 100% |
| Barbary sheep |  | *R. raoultii* | 99.6% |
| Wild boar |  | *R. massiliae* | 100% |
| Common kestrel |  | *R. aeschlimannii* | 99.8% |
| Goshawk |  | *R. massiliae* | 100% |
| Spanish ibex |  | *R. massiliae* | 100% |
| Hare |  | *R. massiliae* | 100% |
| Dog |  | *R. massiliae* | 100% |
| Hedgehog |  | *R. massiliae* | 99.6% |
| Barbary sheep |  | *R. massiliae* | 100% |
| Hedgehog |  | *R. massiliae* | 100% |
| Dog |  | *R. massiliae* | 99.8% |
| **Host species** | **Tick species** | ***Borrelia* species** | **Homology (%)** |
| Dog | *R. sanguineus* | *B. afzelii/B. garinii* | 100% |
| Tortoise |  | *B. afzelii/B. garinii* | 100% |
| Tortoise | *H. lusitanicum* | *B. afzelii/B. garinii* | 100% |
| Barbary sheep | *I. ricinus* | *B. afzelii/B. garinii* | 100% |
| Barbary sheep |  | *B. afzelii/B. garinii* | 100% |
